# Apoptosis-induced ectodomain shedding of hypoxia-regulated carbonic anhydrase IX from tumor cells: a double-edged response to chemotherapy

**DOI:** 10.1186/s12885-016-2267-4

**Published:** 2016-03-19

**Authors:** Ivana Vidlickova, Franck Dequiedt, Lenka Jelenska, Olga Sedlakova, Michal Pastorek, Stanislav Stuchlik, Jaromir Pastorek, Miriam Zatovicova, Silvia Pastorekova

**Affiliations:** Department of Molecular Medicine, Institute of Virology, Biomedical Research Center, Slovak Academy of Sciences, Dubravska cesta 9, 845 05 Bratislava, Slovak Republic; Department of Molecular Biology, Faculty of Natural Sciences, Comenius University in Bratislava, Bratislava, Slovakia; Cellular and Molecular Biology Unit, Gembloux Agro-Bio Tech, University of Liege, Liege, Belgium; Regional Centre for Applied Molecular Oncology, Masaryk Memorial Cancer Institute, Brno, Czech Republic

**Keywords:** Carbonic anhydrase IX, Apoptosis, Hypoxia, Ectodomain, Metalloproteinase, Shedding, Chemotherapy

## Abstract

**Background:**

Carbonic anhydrase IX (CA IX) is a tumor-associated, highly active, transmembrane carbonic anhydrase isoform regulated by hypoxia and implicated in pH control and adhesion-migration-invasion. CA IX ectodomain (ECD) is shed from the tumor cell surface to serum/plasma of patients, where it can signify cancer prognosis. We previously showed that the CA IX ECD release is mediated by disintegrin and metalloproteinase ADAM17. Here we investigated the CA IX ECD shedding in tumor cells undergoing apoptosis in response to cytotoxic drugs, including cycloheximide and doxorubicin.

**Methods:**

Presence of cell surface CA IX was correlated to the extent of apoptosis by flow cytometry in cell lines with natural or ectopic CA IX expression. CA IX ECD level was assessed by ELISA using CA IX-specific monoclonal antibodies. Effect of recombinant CA IX ECD on the activation of molecular pathways was evaluated using the cell-based dual-luciferase reporter assay.

**Results:**

We found a significantly lower occurrence of apoptosis in the CA IX-positive cell subpopulation than in the CA IX-negative one. We also demonstrated that the cell-surface CA IX level dropped during the death progress due to an increased ECD shedding, which required a functional ADAM17. Inhibitors of metalloproteinases reduced CA IX ECD shedding, but not apoptosis. The CA IX ECD release induced by cytotoxic drugs was connected to elevated expression of CA IX in the surviving fraction of cells. Moreover, an externally added recombinant CA IX ECD activated a pathway driven by the Nanog transcription factor implicated in epithelial-mesenchymal transition and stemness.

**Conclusions:**

These findings imply that the increased level of the circulating CA IX ECD might be useful as an indicator of an effective antitumor chemotherapy. Conversely, elevated CA IX ECD might generate unwanted effects through autocrine/paracrine signaling potentially contributing to resistance and tumor progression.

## Background

Carbonic anhydrase IX (CA IX) belongs to a carbonic anhydrase family of enzymes that use a zinc-activated hydroxide mechanism to catalyze the reversible conversion of carbon dioxide to carbonic acid in the net reaction CO_2_ + H_2_O ↔ HCO_3_^−^ + H^+^ [1, 2]. Via this catalytic activity, carbonic anhydrases either supply bicarbonate for biosynthetic reactions and ion transport across membranes or consume produced/transported bicarbonate. Their proper performance is essential for various physiological processes in virtually all organisms. Human CAs exist in 15 isoforms that differ by subcellular localization and kinetic properties [3].

In contrast to other CA isoforms that are mostly present in differentiated cells, expression of CA IX is limited to only few normal tissues, mainly the epithelia of gastrointestinal tract [4]. On the other hand, CA IX is closely associated with a broad range of tumors that either suffer from hypoxia or contain inactive pVHL [5]. This expression pattern is principally determined by a strong HIF-1-mediated transcriptional activation of the *CA9* gene, which contains an HRE element localized on the negative DNA strand immediately upstream of the transcription start site [5].

Despite the dramatic induction by hypoxia, intratumoral distribution of the CA IX protein only partially overlaps with the distribution of low p0_2_ measured by microelectrodes and with the distribution of other markers of hypoxia, such as pimonidazole, HIF-1α, GLUT-1 and VEGF. This can be explained by the high post-translational stability of the CA IX protein, which reflects both actual and expired hypoxia [6], and by its regulation by other microenvironmental factors, such as acidosis [7] and/or by shedding of the extracellular domain of CA IX [8, 9].

CA IX is primarily expressed as a transmembrane protein localized on the surface of tumor cells, where it contributes to regulation of pH through facilitation of bicarbonate transport to the cytoplasm for intracellular alkalinization and to production of protons in the pericellular space for microenvironmental acidosis [10, 11]. CA IX also supports cell adhesion and spreading, and promotes epithelial-mesenchymal transition through stimulation of cell migration and invasion [12, 13]. These attributes of CA IX determine its role in the protection of tumor cells from hypoxia and acidosis.

About 10 % of the cell-associated CA IX molecules undergo constitutive ectodomain (ECD) shedding, which is sensitive to the metalloproteinase inhibitor batimastat. This basal ECD release can be several-fold induced by the treatment with PMA and pervanadate and the induction depends on the presence of ADAM17, a disintegrin and metalloproteinase also called the TNF-α converting enzyme [9]. Thus, the cleavage of the CA IX ECD appears to be a regulated process that responds to signal-transduction stimuli and may contribute to the adaptive changes in the protein composition of tumor cells and of their microenvironment.

A growing number of experimental and clinical studies have demonstrated correlations of CA IX expressed in tumor or stromal cells to aggressive phenotype, resistance to chemo-/radiotherapy and poor cancer prognosis in a spectrum of tumor types [14]. On the other hand, potential clinical value of the CA IX ectodomain is not so clear. While certain studies support its prognostic/predictive value, others fail to find any significant relationship between the CA IX ECD levels and clinical parameters [15–23]. These controversial data may be caused by the use of different detection assays [24], but also by poor understanding of the clinically relevant signals contributing to induction of the CA IX ECD release and its biological consequences.

Here we studied the effect of a cytotoxic drug treatment on the shedding of the CA IX ECD and found that the level of the CA IX ECD is increased in response to induction of apoptosis by inhibition of proteosynthesis, as well as by treatment with the chemotherapeutic drug doxorubicin. Our data suggest that the production of CA IX ECD is a consequence of cell death and imply that the ECD released from tumor cells can either indicate cytotoxic effect of chemotherapy or mediate signaling that promotes cancer development.

## Methods

### Cell culture

CGL3 hybrid cell line was generated by fusion of cervical carcinoma HeLa cells with normal human fibroblasts [25]. HeLa cells with endogenous, hypoxia-inducible expression of CA IX, and MDCK-CA9 cells transfected with the full-length CA9 cDNA and exhibiting constitutive CA IX expression were described earlier [10]. CHO-wt and shedding-defective CHO-M2 cells (with inactive ADAM17) were generously provided by prof. Joaquin Arribas (Vall d’Hebron Institute of Oncology, Barcelona) [26]. The cells were grown in DMEM supplemented with 10 % FCS under standard conditions. Experiments in hypoxia (2 % O_2_) were done in an anaerobic workstation (Ruskinn Technologies) with 5 % CO_2_, 10 % H_2_ and 83 % N_2_ at 37 °C.

### Inhibitors and drugs

Metalloproteinase inhibitor BB-94 (batimastat; British Biotechnology Ltd), Z-VAD-FMK (caspase inhibitor IV, Calbiochem), cycloheximide (CHX), doxorubicin (DOX), actinomycin D (ActD), dexamethasone (DX), phorbol 12-myristate 13-actetate (PMA, all from Sigma-Aldrich) were dissolved in dimethyl sulphoxide and stored in aliquots at −20 °C. Prior to use, the inhibitors were diluted in culture medium to working concentrations (10 μM BB-94, 10 μM PMA, 20–100 μg/ml CHX, 1 μM Z-VAD-FMK, 10 μM DX, 2 μg/ml ActD, and 1 μg/ml DOX).

The cells were plated at a density of 30–50 000 cells/cm^2^, and incubated for 24 h at 37 °C. The treatment periods varied for individual agents, for CHX, ActD, DX and PMA it was 3–6 h, for DOX it was 24 h at 37 °C in normoxic or hypoxic conditions. Parallel control dishes were maintained in the same conditions for the same time periods.

For inhibition of shedding, CGL3 cells were plated and allowed to attach for 24 h at 37 °C, and then incubated for additional 24 h in normoxia or hypoxia. After 1 h incubation in fresh serum-free DMEM medium, the cells were treated with BB-94 inhibitor for 1.5 h, and with CHX for additional 3 h at 37 °C in normoxia or hypoxia.

Assay with the caspase inhibitor Z-VAD-FMK was performed using HeLa cells plated, allowed to attach overnight and incubated for 24 h in hypoxia to induce the expression of CA IX. Then the medium was replaced with fresh serum-free medium containing 1 μM caspase inhibitor Z-VAD-FMK. Six hours later, doxorubicin was added at a final concentration of 1 μg/ml and the cells were incubated for additional 20 h. The cells were harvested for qPCR and FACS analyses and media were collected for ECD assessment by ELISA.

### Antibodies

The primary antibodies were as follows: M75 and IV/18 anti-human CA IX mouse monoclonal antibodies recognizing the N-terminal proteoglycan-like domain and V/10 mouse monoclonal antibody specific for the extracellular catalytic domain were described earlier [27]. Secondary anti-mouse antibodies conjugated with horse-raddish peroxidase were purchased from Sevapharma, donkey anti-mouse IgG Alexa Fluor 488 or 594 were from Invitrogen, and horse anti-mouse FITC-conjugated antibody from Vector Laboratories.

### Quantitative real-time PCR (qPCR)

Total RNA was extracted using the Instapure reagent (Eurogentec). RNA was transcribed with a High-Capacity cDNA Reverse Transcription Kit (Applied Biosystems) using random heptameric primers. Quantitative real-time PCR was performed on a StepOne Real-Time PCR System (Applied Biosystems) using POWER SYBR Green PCR Master Mix (Applied Biosystems) and the following primers: CA9 sense: 5′-TATCTGCACTCCTGCCCTCTG-3′ and CA9 antisense: 5′-CACAGGGTGTCAGAGAGGGTG-3′; Nanog sense: 5′-GCAAATGTCTTCTGCTGAGATGC-3′ and Nanog antisense: 5′-AGCTGGGTGGAAGAGAACACAG-3′; β-actin sense: 5′-TCCTCCCTGGAGAAGAGCTA-3′ and β-actin antisense: 5′-ACATCTGCTGGAAGGTGGAC-3′.

### Cloning and purification of the recombinant CA IX ECD

DNA fragment encoding the extracellular part of the CA IX protein (aa 38–406) was cloned in the pSecTag2A plasmid and expressed as a recombinant fusion protein HisTag-CA IX ECD (rCA IX ECD). The plasmid-transfected HEK 293T cells were cultured in BelloCell® Culture System (Chemglass). Production of rCA IX ECD was done in the EX-CELL 293 serum-free medium (SAFC Biosciences). Conditioned medium was dialyzed and rCA IX ECD was purified by FPLC purification on Ni-precharged HisTrap HP column, concentrated by dialysis against PBS, and stored at −20 °C. The protein was diluted 10^5^-times in culture medium (to 10 ng/ml) immediately before addition to cells.

### Immunoblotting

Protein extraction, SDS-PAGE separation, blotting and immunodetection of CA IX were performed as described earlier [27].

### Enzymed-linked immunosorbent assay (ELISA)

The capture antibody V/10 (10 μg/ml) was immobilized on the surface of microplate wells overnight at 4 °C. After blocking and washing, cell extracts or media diluted in PBS were added to the coated wells for 2 h at RT. The attached antigen was then allowed to react with the mixture of biotinylated MAbs M75 and IV/18 diluted 1:7500 (200 ng/ml) in the blocking buffer. The amount of bound detector antibodies was determined after 1 h incubation with peroxidase-conjugated streptavidin (Pierce) using the peroxidase substrate orthophenylene diamine (Sigma).

### Immunofluorescence

Cells grown on glass coverslips were washed with PBS and fixed in ice-cold methanol at −20 °C for 5 min. Nonspecific binding was blocked with PBS containing 1 % BSA for 30 min at 37 °C. Then, the cells were incubated with the M75 antibody for 1 h at 37 °C followed by anti-mouse FITC-conjugated horse antibody (Vector Laboratories) diluted 1:300 in PBS–BSA for 1 h at 37 °C. The nuclei were stained with propidium iodide (Sigma). The coverslips were mounted onto slides in the Fluorescent Mounting Media (Calbiochem), and the cells were analyzed with Leica DM4500B microscope and photographed with Leica DFC480 camera.

### Evaluation of apoptosis

Internucleosomal cleavage was visualized by the extraction of small detergent-soluble fragments of DNA in 0.5 % Triton X-100, followed by electrophoresis in a 1.5 % agarose gel. Alternatively, apoptotic cells were identified using the terminal deoxynucleotidyltransferase-mediated dUTP-biotin nick end labeling (TUNEL) method, as described previously [28]. Furthermore, the cells were fixed in ice-cold methanol, labeled using M75 MAb for 30 min at 4 °C followed with the FITC-conjugated secondary antibodies and stained by propidium iodide in the presence of RNAse A as described below.

Apoptotic cells were quantified by FACS analysis of the subdiploid DNA content in parallel with the detection of cell surface CA IX. The cells were scraped from the dishes into culture medium, centrifuged at low speed, washed twice with PBS, incubated for 30 min at 4 °C with the M75 monoclonal antibody, centrifuged, washed twice with PBS containing 1 % FCS and incubated with the secondary anti-mouse Alexa Fluor 488 antibody for 30 min at 4 °C. Then the cells were fixed in ice-cold 70 % ethanol for 60 min at −20 °C. Pelleted cells were rehydrated in PBS, resuspended in 200 μl of RNase A solution (5 μg/ml RNase A, 0.1 % Tween 20 in PBS) and incubated for 20 min at 37 °C. Eight hundred microliters of propidium iodide (20 μg/ml stock concentration; diluted 1:250 in PBS) was added to the cell suspension and incubated for 5 min at 4 °C. Analysis was performed on a Becton Dickinson FACScan or Guava easyCyte 6HT (Millipore) flow cytometers. Data were analyzed with Cellquest software (Becton Dickinson Immunocytometry) or with Guava Soft™ software version 1.1 (Millipore). Debris, cell doublets and clumps were excluded from analyses by scatter gating, and a total of 10,000 single cells were analyzed for each sample. Cells with less than the 2 N amount of DNA were classified as apoptotic.

For the comparative viability analysis of the CA IX- versus CA IX+ cells, the CGL3 cells were first labeled with M75 and anti-mouse Alexa Fluor 594 antibody, then incubated with TO-PRO*-3 iodide and sorted using the FACSAriaTM III sorter (Beckton Dickinson). TO-PRO*-3-positive dead cells were excluded and the subpopulations of cells expressing and non-expressing CA IX (25 % of the highest and lowest M75 staining), respectively, were plated, allowed to attach for 24 h, and treated with CHX as described above. The cells were harvested (at 1 × 10^6^ cells/sample), washed in PBS, labeled and analyzed by the FACSCanto™ II flow cytometer (Beckton Dickinson) equipped with the 488 and 633 nm lasers for dye excitation. Labeling of the viable cells was performed in PBS with 10 nM fluorescein diacetate (FDA) for 25 min at room temperature in the dark followed by propidium iodide (PI) at a final concentration of 5 μg/ml. Emitted fluorescence was collected using the 530/30 filter for FDA and 585/42 filter for PI. Viable populations showed positive staining for FDA and negative staining for PI. Data were analyzed with the FCS Express version 4.0 (De Novo Software).

### Cignal assay

HeLa cells were plated at a density of 40 000 cells per well (96-well plate). Cignal®TM Cell-based Multi-Pathway Activity Assay (SABioscience) was performed according to instructions of the manufacturer. Recombinant rCA IX ECD was added for 48 h at final concentration of 10 ng/ml. Dual-luciferase results were calculated for each transfectant and analyzed by the Data Analysis Software (SABioscience). Changes in the activity of each signaling pathway were determined by comparing the normalized luciferase activities of the reporter in treated versus untreated transfected cells.

### Statistical analysis

Results were analyzed by two-tailed unpaired t test (Student’s test), and *P* < 0.05 was considered significant.

## Results

### Apoptosis induced by cycloheximide preferentially occurs in CA IX-negative cells and reduces CA IX expression

Intratumoral CA IX expression has been associated with poor response to chemotherapy and cancer progression in several tumor types including carcinoma of the cervix uteri [29]. To get insight into this phenomenon, we investigated the relationship between CA IX expression and the response of cancer cells to cytotoxic treatment. As a model for the study of CA IX expression during cell death, we initially decided to use CGL3 cells, which were generated by fusion of HeLa human cervical carcinoma cells with normal human fibroblasts [25]. These cells represent a tumorigenic segregant of a non-tumorigenic parental hybrid cell line CGL1. Whereas CGL1 cells show only very low expression of CA IX in normoxia and strongly induce CA IX in hypoxia, CGL3 cells express relatively high CA IX levels even under normoxic conditions. Moreover, flow cytometry revealed that approximately half of the normoxic CGL3 cells show the cell surface expression of CA IX and that labeling with the CA IX-specific antibody M75 allows for an easy gating and separate analysis of the CA IX-positive and CA IX-negative subpopulations growing together in the same culture dish (see below).

**Fig. 1:**
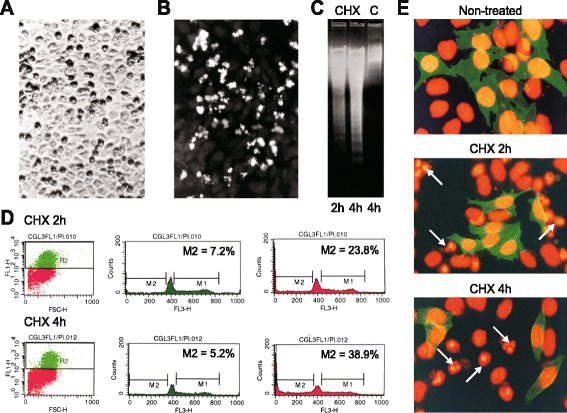
Cycloheximide induces apoptosis in CGL3 cells. **a** Phase-contrast micrograph of the normoxic CGL3 cells treated with 100 μg/ml CHX for 3 h. Dying cells show typical dark granular appearance. **b** Fluorescence image of the CHX-treated CGL3 cells subjected to terminal deoxytransferase-based labeling. The apoptotic cells show nuclear fragments of intense fluorescence. **c** Electrophoretic analysis of the chromosomal DNA fragmentation in CGL3 cells treated with 20 μg/ml CHX for 2 h, 4 h, and absence of apoptotic fragments in the control non-treated cells. **d** FACS analysis of the subG1 cells in subpopulations expressing versus non-expressing CA IX following the treatment with 20 μg/ml CHX for 2 and 4 h and staining with M75 and propidium iodide. **e** Staining of the CHX-treated CGL3 cells for CA IX with the M75 MAb and FITC-conjugated secondary antibody (*green*) and for cell nuclei with propidium iodide (*red*). Fragmented apoptotic nuclei are visible in the CA IX-negative cells (see *arrows*)

One of the cytotoxic agents that can effectively and rapidly trigger apoptosis is cycloheximide (CHX), an inhibitor of proteosynthesis. In CGL3 cells, CHX was able to induce all typical biochemical and morphological features of apoptosis, as visible from the cell morphology in the phase-contrast microscope, where the apoptotic cells appear as dark granular rounded formations compared to flat translucent living cells (Fig. 1a). Induction of apoptosis in response to CHX treatment was also confirmed by the fluorescence analysis of cells using terminal deoxytranferase-based labeling (Fig. 1b) and by the DNA electrophoresis (Fig. 1c). Based on the appearance of typical apoptotic DNA fragments, we could clearly see that the onset of apoptosis is rather fast, proceeding within 2–4 h from the beginning of the CHX treatment.

We then used flow cytometry to quantitatively correlate the progress of cell death with the cell surface expression of CA IX in CGL3 cells. For that purpose, we performed a double labeling of the CHX-treated cells. The cells were first stained with the M75 antibody followed by the FITC-conjugated secondary antibody, then fixed and incubated with propidium iodide to stain DNA for the detection of subG1 signal corresponding to fragmented DNA. After data acquisition, we performed an analysis of the gated CA IX-positive versus CA IX-negative cells to determine a percentage of the hypodiploid cells in each subpopulation. Interestingly, we found considerably higher percentage of apoptotic cells in the CA IX-negative cell subpopulation, i.e. 9 ± 2 % after 1 h treatment with 100 μg/ml CHX, 24 ± 5 % after 2 h treatment and 39 ± 5 % after 3 h treatment compared to the CA IX-positive cells that showed 4 ± 2 % of apoptotic cells after 1 h, 5 ± 3 % after 2 h and 7 ± 2 % after 3 h. Similar difference was observed in CGL3 cells treated with lower concentration of CHX (20 μg/ml) that resulted in decelerated progress of apoptosis (Fig. 1d). These findings suggest that the presence of the cell-surface CA IX was associated with a decreased sensitivity to cycloheximide and a prolonged cell survival. Double immunofluorescence labeling of the CHX-treated CGL3 cells was in line with the above data, since the apoptotic nuclei were visible only among the CA IX-negative CGL3 cells (Fig. 1e).

**Fig. 2:**
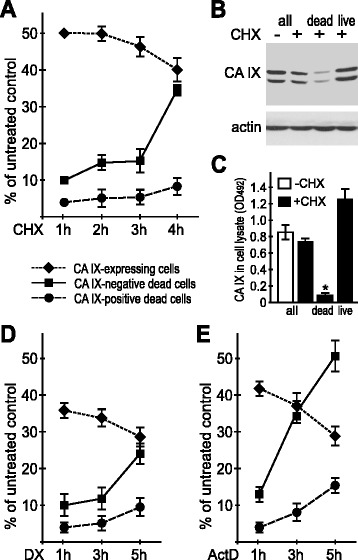
Progression of apoptosis is associated with reduced CA IX expression in CGL3 cells. **a** FACS analysis of the normoxic CGL3 cells treated with 20 μg/ml CHX and stained for CA IX and DNA content as described in Fig. 1d. Graph shows the percentage of the subG1 apoptotic cells in the CA IX-negative and the CA IX-positive subpopulations and changes in the percentage of the CA IX-positive cells. **b** Immunoblotting analysis of the CA IX level before (−) and after the CHX treatment (+) in whole cell population, and in the cells that died or survived the CHX treatment. **c** ELISA assessment of the relative CA IX levels in the CHX-treated CGL3 cells. Error bars represent the mean ± SD,*p < 0.05. **d**, **e** FACS analyses of the CGL3 cells treated with actinomycin D (ActD) and dexamethasone (DX), stained as in Fig. 1d. The data show reduced proportion of the CA IX-positive cells in the course of the treatment and faster progression of apoptosis in the CA IX-negative cells

However, we noticed that the percentage of the CA IX-positive cells decreased during the progression of cell death (Fig. 2a). We verified this observation by the immunoblotting evaluation of the CA IX levels before and after the CHX treatment, which clearly showed reduced amount of the CA IX protein in the lysates from CHX-treated cells (Fig. 2b). Moreover, we separated dead and living CGL3 cells by gentle washing out of the dying cells that do not adhere to support and then scraping off the adherent living cells. These separated subpopulations were then analyzed by immunoblotting for the expression of CA IX. As expected, we found much lower CA IX level in the dying cells when compared to the living ones (Fig. 2b). This was confirmed by the ELISA assessment of the cell-associated CA IX in dead versus living cells (Fig. 2c). Similar results were obtained with the CGL3 cells treated with actinomycin D and dexamethasone, respectively (Fig. 2d, e).

### CA IX-negative subpopulation of CGL3 cells is more susceptible to apoptosis

**Fig. 3:**
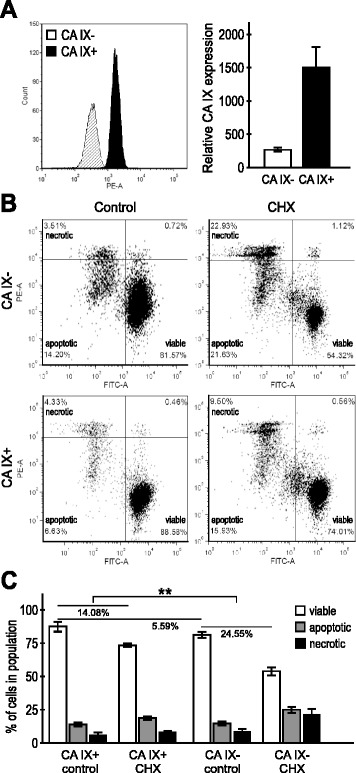
CA IX-negative CGL3 cells are more susceptible to apoptosis. **a** Representative FACS analysis of the CA IX expression in the sorted CA IX-negative (CA IX-) versus CA IX-positive (CA IX+) CGL3 cell subpopulations, and relative CA IX expression in the sorted subpopulations from four independent experiments. **b** Representative FACS analysis of the cell viability in the sorted subpopulations using double staining with the fluorescein di-acetate and propidium iodide. **c** Proportion of the viable, apoptotic and necrotic cells in the sorted subpopulations under control conditions and after treatment with CHX, as determined by FACS of FDA-PI staining in four independent experiments. CHX-treated CA IX cells showed significantly higher viability than CA IX- cells. Error bars represent the mean ± SD. **p < 0.01

To demonstrate that CA IX expression is linked to better cell survival, we labeled the normoxic CGL3 cells with the CA IX-specific M75 antibody and sorted the lower 25 % of M75-negative CA IX- subpopulation and the upper 25 % of M75-positive CA IX+ subpopulation (Fig. 3a). Then we treated these subpopulations with CHX and compared their survival based on the sequential staining with fluorescein di-acetate (FDA), which emits fluorescence in living cells, followed by propidium iodide, which accumulates in late apoptotic and necrotic cells depending on the degree of their membrane disruption. Indeed, we found a significantly lower proportion of viable cells and a higher proportion of both apoptotic and necrotic cells in the CA IX- subpopulation than in the CA IX+ counterpart (Fig. 3b, c), supporting the view that the expression of CA IX is associated with reduced vulnerability to cell death.

### Loss of CA IX in dying cells is sensitive to metalloproteinase inhibition

**Fig. 4:**
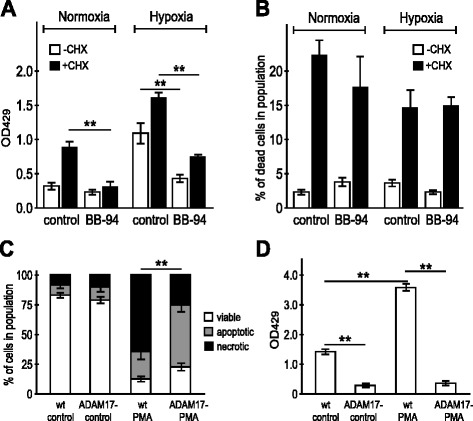
Inhibition of metalloproteinases reduces shedding, but does not block apoptosis. **a** ELISA detection of the CA IX ECD in the culture media of CHX-treated versus non-treated CGL3 cells in the presence or absence of the metalloproteinase inhibitor BB-94. BB-94 significantly reduces shedding of the CA IX ECD. **b** FACS analysis of the proportion of dead cells (based on PI uptake) in the corresponding populations of the CHX- and/or BB-94-treated cells compared to non-treated controls. BB-94 showed no significant effect on cell death. **c** FACS analysis of the viability of the ADAM17-competent CHO-wt cells (wt) versus ADAM17-defective CHO-M2 cells (ADAM17-) treated or not by PMA. Slower progression of cell death can be seen in ADAM17- cells. **d** ELISA detection of the shed CA IX ECD from the same CHO cell cultures. Data confirm that CA IX ECD shedding is ADAM17-dependent. Error bars represent the mean ± SD. **p < 0.01, *p < 0.05

In accord with the known phenomenon of global proteolytic release of cell surface proteins associated with apoptosis [30], we decided to examine the possibility that the CA IX ECD is increasingly cleaved from the dying cells. Level of the shed CA IX ECD was assessed using sandwich ELISA with the CA IX-specific monoclonal antibodies binding to the N-terminal PG-like domain and the catalytic CA domain [9]. We found that the CHX-treated cells produced significantly higher CA IX ECD levels when compared to non-treated cells (Fig. 4a). Similar increase was observed under both normoxia and hypoxia, although the total extracellular level of CA IX ECD was higher in hypoxic cells independently of the CHX treatment.

We previously showed that the CA IX shedding is a metalloproteinase-dependent process, which can be reduced by the metalloproteinase inhibitor batimastat, i.e. BB-94 [9]. In agreement with this earlier finding, pre-treatment of the cells with BB-94 led to a decreased level of the CA IX ECD in the culture medium of the CHX-treated cells supporting the idea that induction of apoptosis led to the activation of metalloproteinases involved in the shedding of the CA IX ECD (Fig. 4a). Noteworthy, BB-94 itself had no significant effect on the extent of cell death (Fig. 4b).

In addition, we evaluated the viability of ADAM17-competent CHO-wt versus ADAM17-defective CHO-M2 cells transiently expressing CA IX and treated with PMA (since these cells are not sensitive to CHX). We found that the ADAM17-competent cells, which shed CA IX, displayed reduced viability in response to PMA when compared to the ADAM17-defective cells, which are unable to cleave the CA IX ectodomain to culture medium (Fig. 4c,d).

### Doxorubicin induces CA IX ECD shedding

Next we wanted to test whether clinically used chemotherapeutic drugs represented here by doxorubicin could also trigger the CA IX ECD shedding from the dying cancer cells. In addition to CGL3 cells, we also included HeLa cells as well as MDCK-CA9 transfectants, which express CA IX protein independently of hypoxia and other pathways regulating the transcription of the *CA9* gene, and thus allow for reliable evaluation of the efficacy of the shedding itself. The cells were incubated for 24 h in normoxia and hypoxia, respectively, in the absence or presence of 1 μg/ml doxorubicin (DOX), collected and stained first with the M75 antibody to detect the cell surface CA IX, then fixed and incubated with propidium iodide to stain DNA. The cells were analyzed by flow cytometry to determine % of CA IX-positive cells and % of subG1 cells undergoing apoptosis. Simultaneously, we collected the culture media of the analyzed cells and used it for the determination of the shed CA IX ECD level.

**Fig. 5:**
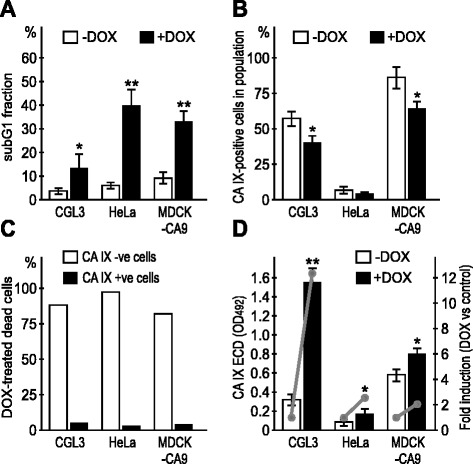
Doxorubicin-induced apoptosis is associated with reduced CA IX expression and increased CA IX ECD shedding in normoxic cells. **a** FACS analysis of the subG1 fraction in the control and DOX-treated cells determined by the DNA labeling with propidium idodide, **b** and corresponding proportions of the M75-labeled CA IX-positive cells in the same cell samples. **c** Percentage of the CA IX- and CA IX+ cells within the DOX-treated dead cells. Data show that the dying cells are principally CA IX-negative. **d** Increased levels of the CA IX ECD shed from the DOX-treated versus control normoxic cells. *Grey lines* illustrate the DOX-related fold induction of the CA IX ECD shedding. Error bars represent the mean ± SD. **p < 0.01, *p < 0.05

Consistent with the data obtained above with CHX, we could observe that the increased apoptotic death in DOX-treated cells was associated with the decreased cell-surface expression of CA IX (Fig. 5a, b). This was accompanied by increased CA IX shedding and elevated ECD in the culture medium (Fig. 5d). Moreover, apoptosis was preferentially triggered in the CA IX-negative subpopulation supporting the view that cells eliminate CA IX though shedding of its ECD at the onset or early during cell death (Fig. 5c). Since the experiment was performed in normoxia, HeLa cells showed only a weak CA IX expression and low percentage of the CA IX-positive cells in the population. On the other hand both CGL3 cells and MDCK-CA9 cells displayed relatively high CA IX expression.

**Fig. 6:**
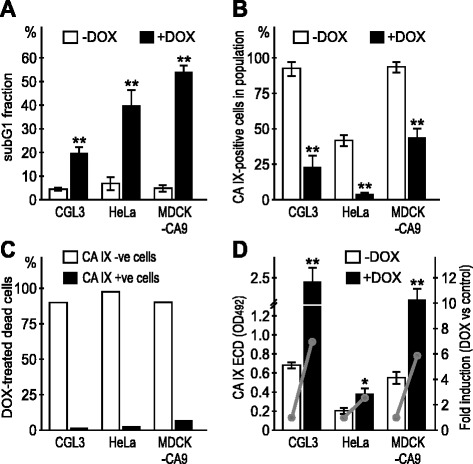
Doxorubicin-induced apoptosis and CA IX ECD shedding in hypoxic cells. **a** FACS analysis of the subG1 fraction in the control and DOX-treated cells exposed to hypoxia (2 % oxygen), (**b**) and corresponding proportions of the M75-labeled CA IX-positive cells in the same cell samples. **c** Percentage of the CA IX- and CA IX+ cells within the DOX-treated dead cells. **d** Absolute levels and fold induction of the CA IX ECD shed from the DOX-treated and control hypoxic cells. Hypoxia induces CA IX expression and boosters the effects of DOX on apoptosis and shedding. Error bars represent the mean ± SD. **p < 0.01, *p < 0.05

When exposed to hypoxia, all cell lines, including HeLa, expressed CA IX levels in an increased percentage of cells in culture when compared to normoxic cells (Fig. 6). Again, apoptosis was confined to the CA IX-negative subpopulation of cells and was accompanied by more dramatic loss of cell surface CA IX and higher ECD shedding.

**Fig. 7:**
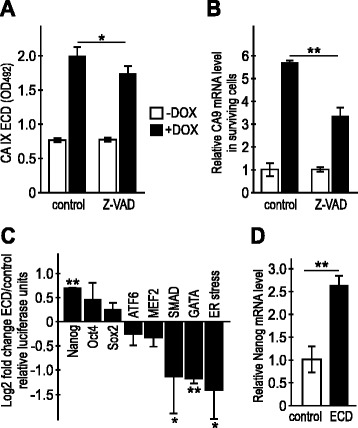
Pan-caspase inhibitor Z-VAD-FMK (Z-VAD) reduces CA IX shedding and expression in hypoxic HeLa cells, and recombinant CA IX ECD induces the Nanog pathway. **a** ELISA of the CA IX ECD shed from the whole population of the DOX and Z-VAD-FMK-treated cells. **b** qPCR analysis of the CA IX transcript level in the viable fraction of cells. DOX-surviving cells show higher ECD shedding and higher transcription of CA IX, whereas Z-VAD inhibits the ECD release and reduces CA IX transcription. **c** Cell-based dual luciferase reporter assay of HeLa cells incubated in the presence of recombinant rCA IX ECD showed alterations of several signal transduction pathways leading to changes in transactivation activities of the transcription factors indicated in the graph. **d** qPCR analysis confirmed the induction of Nanog gene transcription in the rCA IX ECD-treated cells. Error bars represent the mean ± SD. **p < 0.01, *p < 0.05

We also wanted to see, whether inhibition of the executors of apoptosis can affect the CA IX ECD shedding. Therefore, we treated HeLa cells with the pan-caspase inhibitor Z-VAD-FMK. Since CA IX expression in HeLa cells depends on hypoxia, we first incubated the cells in hypoxia for 24 h, then added the inhibitor followed by DOX 6 h later. The culture medium was harvested after 20 h and analyzed for the CA IX ECD (Fig. 7a). As expected, the pan-caspase inhibitor Z-VAD-FMK decreased the level of the CA IX ECD in the DOX-treated cells, suggesting that the execution of apoptosis contributes to the induction of CA IX cleavage. Interestingly, the DOX-increased shedding was associated with the significantly elevated CA IX transcription in the cells surviving the treatment, but this elevation was partially blunted by the pan-caspase inhibitor Z-VAD-FMK, suggesting a feedback regulation by which shedding-related CA IX loss from the cell surface may be compensated by its de novo synthesis (Fig. 7b).

### CA IX ECD contributes to intracellular signaling

The above-described transcriptional changes in the expression of CA IX prompted us to investigate potential signaling effects of the externally added CA IX ECD. To this end we performed the CIGNAL analysis of the normoxic HeLa cells in the presence versus absence of the recombinant CA IX ECD (rCA IX ECD). Since under normoxia, HeLa cells show no or only very weak expression of endogenous CA IX, they release no or low CA IX ECD and are thus suitable for such analysis. Exposure of HeLa cells to rCA IX ECD at 10 ng/ml concentration (that is achievable in human sera [18]) led to the activation of the pathway executed by the Nanog transcription factor implicated in epithelial-mesenchymal phenotype and stemness [31]. On the other hand, pathways driven by the transcription factors mediating stress responses or tumor suppression, including GATA, SMAD2/3/4 and MEF2 were dowregulated [32–34], (Fig. 7c). Interestingly, qPCR analysis demonstrated significantly increased transcriptional levels of Nanog in the ECD-treated HeLa cells, supporting the CIGNAL data (Fig. 7d). These findings indicate that the CA IX ECD can participate in autocrine/paracrine signaling to cancer cells and contribute to transcription of genes implicated in tumor phenotype. Nevertheless, tumor milieu contains many other soluble factors that may accentuate or attenuate the CA IX ECD signaling.

## Discussion

Ectodomain shedding is an important molecular mechanism regulating cell surface density and/or activity of a number of transmembrane proteins implicated in signal transduction, such as receptors (EGFR, TNRF, IL-6R, Notch), adhesion molecules (CD44, VCAM, cadherins), growth factors and cytokines (HB-EGF, TGFα, TNFα, IL-6) [35]. It occurs constitutively at low basal levels, but can be strongly induced in response to diverse stimuli including cytotoxic stress associated with anticancer therapy. Chemotherapy-induced apoptosis elevates transcription and activates ADAM17, a principal metalloproteinase that orchestrates shedding and pro-survival responses in cancer cells through the release of factors that enhance tumor growth via autocrine and/or paracrine signaling [36–38].

Here we showed that CA IX, a hypoxia-induced protein expressed on the surface of tumor cells exhibits increased ectodomain shedding from cells undergoing apoptosis induced by cytotoxic compounds. This phenomenon can be observed as an acute response to the inhibitor of macromolecular synthesis cycloheximide, as well as a response to the chemotherapeutic drug doxorubicin. Similar effects on CA IX ECD shedding were obtained with actinomycin D and dexamethasone. This apoptosis-associated shedding can be inhibited with a broad metalloproteinase inhibitor batimastat in agreement with the fact that CA IX is an ADAM17 substrate [9]. In contrast, BB-94 cannot block the onset of apoptosis indicating that shedding is not the prerequisite, but rather a consequence of apoptosis. This is in line with other recently published studies [37, 38] and also with our observation that the pan-caspase inhibitor Z-VAD-FMK can reduce the CA IX ECD shedding.

These data may indicate that the elevated CA IX ECD in the blood of tumor patients can result from efficient chemotherapy leading to cell death and tumor shrinkage. Moreover, taking into account the role of the plasma membrane CA IX in the protection of tumor cells from hypoxia and acidosis and in migration-invasion, we might speculate that reducing the surface density of CA IX through its ECD shedding could make tumor cells less invasive and more vulnerable to microenvironmental stresses. Albeit so far there is only one study supporting the role of ECD (actually, of an increased post-treatment ECD level) as an indicator of a good outcome of neoadjuvant chemotherapy [39], there are dozens of clinical papers showing that the high cell-surface expression of CA IX is linked to chemoresistance and a more aggressive tumor phenotype [14].

However, the situation is not so simple (1) because elevated shedding in dying subpopulation of cells leads to an increased CA IX expression in the surviving subpopulation of cells and (2) because ECD itself can induce transcription of genes implicated in stem-like, pro-metastatic phenotype through Nanog mRNA elevation and transactivation of the Nanog-driven pathways. Translating these findings into tumor biology would suggest that the cytotoxic treatment might preferentially kill subpopulation of cells, which do not express CA IX or which lose CA IX through elevated ECD shedding, and spare those cells that retain CA IX on their surface. At the same time, ECD released from the dying cells might signal to surviving cells and affect their phenotype through modulation of their transcriptional program. This would then create the basis for the outgrowth and progression of resistant tumor cells. Further translating these assumptions into clinical context, this would mean that high blood level of the CA IX ECD might be an unwanted negative effect of chemotherapy that promotes tumor progression through the CA IX ECD-emitted signaling. Mechanism(s) behind this signaling remain to be elucidated, but it is quite conceivable that the ECD binds to a so far unknown receptor and thereby mediates signal transduction to intracellular pathways, such as the one driven by Nanog. Interestingly, MatInspector-based in silico analysis of the *CA9* promoter showed that it contains a putative binding site for the Nanog transcription factor (-89/-107 position, matrix similarity 0.959) thus offering an explanation for the feedback regulation of the *CA9* transcription via CA IX ECD [40].

This scenario is compatible with several reports linking high CA IX expression in tumors and/or high CA IX ECD levels with resistance to chemotherapy (including doxorubicin), increased survival of tumor cells, stem-like phenotype and poor prognosis of tumor patients [15–19, 41–44]. It is also supported by experimental studies showing that the CA IX expressing cells are viable, clonogenic and resistant to killing by ionizing radiation [45, 46], in line with our findings of the reduced cell death in the CA IX-positive cells and increased CA IX expression in the cells surviving the cytotoxic treatment. Moreover, CA IX expression has been implicated in activation of stromal fibroblasts and their crosstalk with prostate cancer cells resulting in the increased cancer invasiveness [47]. Although the authors do not consider a role for CA IX shedding, it cannot be excluded that the observed pro-metastatic effects are at least partially mediated by the paracrine signaling of the CA IX ECD. Last but not least, induction of EMT-related pathways/genes (such as Nanog) observed here is in line with earlier data showing that CA IX ECD can inhibit cell adhesion [48] and suggests that it may contribute to detachment and dissemination of cancer cells.

Taking into account these controversial outcomes of the CA IX ECD shedding, it is also well conceivable that in the reports that failed to find any significant relationship of the CA IX ECD levels to clinico-pathological correlates [20–22], we actually face a complex situation, in which some patients may release CA IX ECD as a consequence of a complete response to therapy, whereas others may release CA IX ECD as a partial response to therapy that later resumes in recurrence. This latter subgroup of patients could be potentially identified by detection of CA IX in metastases. Albeit data from such studies are rare, there are few reports showing that CA IX is indeed expressed in metastatic lesions [49–52].

Taking together, here we reveal the CA IX ECD shedding as a response to cell death induced by cytotoxic drugs and propose how it might affect tumor biology and influence the interpretation of clinical data from the assessment of the soluble CA IX levels in cancer patients. Nevertheless, this study provides only initial insight into the potential role of CA IX ECD in cancer development, and additional thorough investigations are needed to better understand this phenomenon.

## Conclusions

Intratumoral expression of the hypoxia biomarker CA IX has been associated with poor response to chemotherapy in many independent studies. Here we showed that: (1) CA IX expression offers survival advantage in cancer cells treated with cytotoxic drugs, (2) induction of apoptosis triggers an increased CA IX ectodomain shedding, (3) CA IX ectodomain contributes to paracrine signaling. These findings support the role of the circulating CA IX ectodomain as an indicator of chemotherapy response and as a signaling molecule implicated in cancer progression.

### Ethics approval and consent to participate

Not applicable.

### Consent for publication

Not applicable.

### Availability of data and materials

The dataset supporting the conclusions of this article is included within the article.

## Abbreviations

ADAM17: a disintegrin and metalloproteinase
BB94: batimastat
CA IX: carbonic anhydrase IX protein
*CA9*: carbonic anhydrase 9 gene
CHX: cycloheximide
DOX: doxorubicin
ECD: extracellular domain
EMT: epithelial-mesenchymal transition
GLUT: glucose transporter
HIF: hypoxia inducible factor
MAb: monoclonal antibody
MMP: metalloproteinase
pVHL: von Hippel Lindau tumor suppressor protein
TNF: tumor necrosis factor
VEGF: vascular epithelial growth factor
